# Is health-related quality of life significantly different in very elderly patients receiving home mechanical ventilation?

**DOI:** 10.36416/1806-3756/e20250230

**Published:** 2026-03-05

**Authors:** Teresa Machado, Tiago Sá, Pedro Viegas, Leonor Roseta, Carla Ribeiro

**Affiliations:** 1. Department of Pulmonology, Unidade Local de Saúde Gaia/Espinho, Vila Nova de Gaia, Portugal.; 2. Department of Community Medicine, Information and Health Decision Sciences (MEDCIDS), Faculty of Medicine, University of Porto, Porto, Portugal.; 3. CINTESIS-RISE Health, Faculty of Medicine, University of Porto, Porto, Portugal.

## TO THE EDITOR,

Home mechanical ventilation (HMV) is widely used to manage chronic respiratory failure of various etiologies; however, challenges persist, particularly among elderly patients, in whom frailty, cognitive impairment, and a higher burden of comorbidities complicate patient selection, ventilator optimization, and long-term adherence.^1^ ^,^ ^2^

Although survival and physiological outcomes are important, health-related quality of life (HRQoL) remains a key measure of therapeutic success. Studies specifically addressing HMV in the very elderly are scarce.^3^ ^,^ ^4^ This population represents an increasing proportion of HMV users, yet it remains unclear whether the benefits observed in younger individuals extend to them. The aim of the present study was to compare HRQoL in HMV patients, focusing on differences between those aged 80 years or older (very elderly) and younger individuals.^5^

We conducted a cross-sectional study involving consenting patients aged 18 years or older who had been treated with HMV for more than three months and were followed at an HMV outpatient clinic. Two validated questionnaires in Portuguese were self-administered by the patients during the clinic visit, with a physician available to provide clarification if needed. The Severe Respiratory Insufficiency (SRI) questionnaire assesses HRQoL in patients with chronic respiratory insufficiency, with scores ranging from 0 to 100, where higher scores indicate better HRQoL.^6^ ^,^ ^7^ The S3 Non-Invasive Ventilation (S3-NIV) questionnaire is a disease-specific tool that evaluates the impact of HMV, with scores ranging from 0 to 10, where higher scores reflect less impact of the disease and its treatment.^8^ ^,^ ^9^ Demographic and clinical data were retrieved from hospital records by the study authors. Data analysis was performed using SPSS Statistics, version 29.0.0 (IBM Corp., Armonk, NY, USA). Categorical variables were expressed as frequencies and percentages, whereas continuous variables were expressed as median (interquartile range [IQR]), as appropriate. Statistical significance was set at p<0.05.

A total of 342 patients were included, of whom 67 (19.6%) were aged ≥80 years. Their characteristics are presented in Table 1, and the questionnaire results are shown in Table 2. Among the described comorbidities, hypertension (77.6% vs. 56.7%, p=0.002) and heart failure (64.2% vs. 30.9%, p<0.001) were significantly more prevalent in patients aged 80 years or older compared to younger individuals. Ventilator settings, adherence, and blood gas parameters were similar between groups.

**Table 1:** Demographic and clinical characteristics of patients aged <80 years and ≥80 years.

	< 80 years 275 (80.4)	≥ 80 years 67 (19.6)	p-value
Age (years)	68.0 (61.0-74.0)	84.0 (82.0-87.0)	<0.001*
Male sex	163 (59.3)	37 (55.2)	0.546
BMI (kg/m^2^)	30.4 (24.1-37.8)	31.1 (25.1-36.6)	0.604
Time under HMV (months)	33.0 (11.0-75.0)	25.0 (9.0-62.0)	0.334
Disease			
COPD	143 (52.0)	31 (46.3)	0.400
OHS	54 (19.6)	22 (32.8)	0.312
RCWD	42 (15.3)	7 (10.4)	0.041*
NMD	24 (8.7)	1 (1.5)	0.020*
ILD	1 (0.4)	2 (3.0)	0.039*
Comorbidities			
Arterial Hypertension	52 (77.6)	156 (56.7)	0.002*
Heart Failure	43 (64.2)	85 (30.9)	<0.001*
Obstructive Sleep Apnea	24 (35.8)	116 (42.2)	0.342
Diabetes mellitus	23 (34.3)	87 (31.6)	0.672
Bronchiectasis	10 (14.9)	50 (18.2)	0.530
Chronic Kidney Disease	8 (11.9)	7 (2.5)	0.003*
Liver Disease	1 (1.5)	12 (4.4)	0.476
IPAP (cm H_2_o)	19.0 (16.0-22.0)	18.0 (16.0-20.4)	0.340
EPAP (cm H_2_o)	6.0 (5.0-8.0)	6.0 (5.0-8.0)	0.107
Respiratory Rate (BPM)	15.0 (14.0-16.0)	16.0 (14.0-16.0)	0.200
Daily usage time (hours/day)	7.0 (6.0-9.0)	8.0 (6.0-11.0)	0.377
pH	7.40 (7.39-7.43)	7.40 (7.38-7.43)	0.960
PaO_2_ (mmHg)	72.4 (65.6-79.0)	70.4 (65.1-76.6)	0.127
PaCO_2_ (mmHg)	46.4 (42.9-50.6)	46.2 (43.1-51.0)	0.915
HCO_3_^-^ (mmHg)	27.8 (26.2-29.6)	27.7 (26.1-29.7)	0.789

HMV, home mechanical ventilation; BMI, body mass index; COPD, chronic obstructive pulmonary disease; RCWD, restrictive chest wall disorders; OHS, obesity hypoventilation syndrome; NMD, neuromuscular disorders; ILD, interstitial lung disease. Results presented as n (%) and median [p25-p75]. p-values were provided by the Mann-Whitney U test and chi-squared tests; *Statistically significant differences between groups (p<0.05).

**Table 2:** Questionnaire results in patients <80 years and ≥80 years.

	< 80 years	≥ 80 YEARS	p-value
SRI N (%)	275 (80.6)	66 (19.4)	
Respiratory complaints	71.9 (50.0-84.4)	68.8 (48.4-84.4)	0.723
Physical functioning	50.0 (33.3-70.8)	33.3 (11.4-63.5)	<0.001*
Attendant symptoms and sleep	60.7 (42.8-75.0)	57.1 (42.8-78.6)	0.607
Social relationship	83.3 (66.7-95.8)	75.0 (58.3-92.7)	0.058
Anxiety	50.0 (35.0-75.0)	60.0 (40.0-85.0)	0.060
Psychological well-being	62.5 (47.2-80.6)	66.7 (38.2-88.9)	0.650
Social functioning	62.5 (46.9-84.4)	56.2 (40.6-75.9)	0.037*
Summary score	63.2 (50.5-74.6)	55.3 (41.6-79.5)	0.294
S3-NIV N (%)	183 (80.6)	44 (19.4)	
Respiratory symptoms	7.0 (5.0-8.0)	6.0 (4.0-8.0)	0.295
Sleep and side effects	7.5 (6.2-8.8)	6.7 (5.4-8.1)	0.007*
Total score	7.3 (5.7-8.2)	6.1 (5.1-7.7)	0.036*

S3-NIV, S3 Non-Invasive Ventilation questionnaire; SRI, Severe Respiratory Insufficiency questionnaire. Results presented as n (%) and median [p25-p75]. p-values were provided by the Mann-Whitney U test; *Statistically significant differences between groups (p<0.05).

Regarding HRQoL, although patients aged ≥80 years had significantly lower scores in physical functioning (33.3 vs. 50.0, p<0.01) and social functioning (56.2 vs. 62.5, p=0.037), no statistically significant differences were observed between groups in the remaining subscales or in the SRI summary scale. The S3-NIV showed similar results in the respiratory symptoms subscale, but significantly lower scores in the sleep and side effects subscale and in the total score among patients ≥80 years (6.7 vs. 7.5, p=0.007; and 6.1 vs. 7.3, p=0.036, respectively).

Differences in the prevalence of underlying diseases were also noted between very elderly and younger patients. While chronic obstructive pulmonary disease and obesity hypoventilation syndrome were similarly common in both groups, restrictive chest wall diseases and neuromuscular disorders were more frequent among the very elderly. However, the small sample sizes in some subgroups limit the ability to draw definitive conclusions. Nonetheless, these findings highlight possible variations in the etiology of respiratory failure across age groups, warranting further investigation.

Consistent with previous studies reporting good tolerance and adherence to HMV in elderly populations, our study also found high daily usage times among very elderly patients.^3^ ^,^ ^4^ As expected with advancing age, these patients had a higher comorbidity burden and lower scores in physical and social functioning. They also reported a greater perceived burden of disease and treatment, particularly related to sleep disturbances and side effects. These findings align with the literature on age-related decline in functional status and increased frailty and may reflect physiological aging processes rather than reduced HMV efficacy.^10^ Moreover, our results are consistent with a recent large cross-sectional study including patients aged ≥75 years, which found similar adherence and efficacy of HMV between older and younger groups, despite the elderly having more comorbidities.^2^ Indeed, in spite of their clinical fragility, daily usage hours were high, suggesting that advanced age and multimorbidity do not necessarily compromise treatment effectiveness.

Evidence specifically focusing on very elderly patients receiving HMV remains limited and methodologically heterogeneous. One study using an age cut-off of 75 years reported significant improvements in quality of life and respiratory function after HMV initiation, despite functional limitations. However, it included a small sample (n=6) and used generic HRQoL tools.^4^ In contrast, our study applied a higher age threshold and disease-specific HRQoL measures, finding no significant differences in HRQoL between age groups. Although changes in HRQoL from the time of HMV initiation were not assessed, the absence of differences between older and younger patients supports the potential of HMV to maintain quality of life even in frailer individuals.

Furthermore, managing HMV in older adults may pose challenges related to cognitive impairment and social support needs. These factors represent important areas for consideration and warrant further research to better understand their impact on care optimization.

This study has some limitations, including its single-center design and the inherent constraints of a cross-sectional approach. First, it can only identify associations between variables rather than establish causality. Second, the findings reflect circumstances at the time of data collection, and the lack of longitudinal follow-up limits the ability to assess changes over time. Third, data collection relied on self-reported information, which may introduce recall bias and affect the accuracy of reported outcomes; however, this limitation is inherent to all self-reported HRQoL instruments. Finally, a formal assessment of cognitive impairment and frailty was not performed in either group. Nevertheless, some indirect information regarding frailty is provided by the physical functioning subscale of the SRI. Moreover, HRQoL assessment is generally challenging in patients with cognitive impairment, and the authors believe this limitation does not significantly affect the study’s conclusions. 

Overall, these results underscore the importance of a personalized approach to HMV management, in which clinical factors take precedence over chronological age, ensuring that very elderly patients receive optimal care despite age-related limitations.
